# Adrenal insufficiency due to bilateral adrenal metastases – A systematic review and meta-analysis

**DOI:** 10.1016/j.heliyon.2019.e01783

**Published:** 2019-05-29

**Authors:** Philippa H. Tallis, R. Louise Rushworth, David J. Torpy, Henrik Falhammar

**Affiliations:** a Division of Medicine, Royal Darwin Hospital, Darwin, NT, Australia; b Department of Medicine, Katherine Hospital, Katherine, NT, Australia; c School of Medicine, Sydney, The University of Notre Dame, Darlinghurst, NSW, Australia; d Endocrine and Metabolic Unit, Royal Adelaide Hospital, Adelaide, SA, Australia; e University of Adelaide, Adelaide, SA, Australia; f Department of Endocrinology, Metabolism and Diabetes, Karolinska University Hospital, Stockholm, Sweden; g Department of Molecular Medicine and Surgery, Karolinska Institute, Stockholm, Sweden; h Menzies School of Health Research, Darwin, NT, Australia

**Keywords:** Oncology

## Abstract

**Objective:**

Bilateral adrenal metastases may cause adrenal insufficiency (AI) but it is unclear if screening for AI in patients with bilateral adrenal metastases is justified, despite the potential for adrenal crises.

**Method:**

A search using PubMed/Medline, ScienceDirect and Cochrane Reviews was performed to collect all original research articles and all case reports from the past 50 years that describe AI in bilateral adrenal metastases.

**Results:**

Twenty studies were included with 6 original research articles, 13 case reports and one case series. The quality was generally poor. The prevalence of AI was 3–8%. Of all cases of AI (n = 25) the mean pooled baseline cortisol was 318 ± 237 nmol/L and stimulated 423 ± 238 nmol/L. Hypotension was present in 69%, hyponatremia in 9% and hyperkalemia in 100%. Lung cancer was the cause in 35%, colorectal 20%, breast cancer 15% and lymphoma 10%. The size of the adrenal metastases was 5.5 ± 2.8 cm (left) and 5.5 ± 3.1 cm (right), respectively. There was no correlation between basal cortisol, stimulated cortisol concentration or ACTH with the size of adrenal metastases. The median time to death was 5.0 months (IQR 0.6–6.5). However, two cases were alive after 12–24 months.

**Conclusion:**

The prevalence of AI in patients with bilateral adrenal metastases was low. Prognosis was very poor. Due to the low prevalence of AI, screening is likely only indicated in patients with symptoms and signs suggestive of hypocortisolism.

## 1 Introduction

Adrenal metastases in patients with malignancy are common, with most data coming from autopsy studies. Between 28-42% of lung cancers and 12–34% of breast cancers metastasize to the adrenals [1, 2], but melanoma, renal, thyroid and colorectal cancer can do so as well [3]. The adrenal glands have a rich sinusoidal supply which could be involved in the pathogenesis [4]. Patients with adrenal metastases have a very poor prognosis and in one study the 2 year survival was only 7% [5], considerably lower than the highly lethal primary adrenocortical carcinoma [6].

Adrenal insufficiency (AI) was first described in 1849 by Thomas Addison who later described AI from adrenal metastases in 1855 [7]. Since then, the prevalence of AI with bilateral metastases has been unclear from the literature despite several studies and an increasing number of case reports documenting the phenomenon.

AI can present with fatigue, weakness, weight loss, anorexia, nausea or vomiting [8]. These symptoms are also commonly seen in metastatic disease. Thus, a high degree of suspicion of AI is required when managing advanced malignancies. However, despite the presence of bilateral adrenal metastases, adrenal function may be preserved until >90% of the cortex is destroyed [9]. Signs of AI include hyperpigmentation from raised adrenocorticotropin hormone (ACTH), hypotension, tachycardia or fever [8]. Biochemically, primary AI may be manifest by hyponatremia, hyperkalemia, hypoglycemia, hypercalcaemia and low cortisol together with elevated ACTH. If AI is not diagnosed, adrenal crisis may supervene, with a potentially fatal outcome [10].

For the diagnosis of primary AI, the ACTH stimulation test is currently regarded as the 'gold standard' [8]. The Endocrine Society Guidelines from 2016 recommend to use 250mcg of ACTH 1–24 (Cosyntropin®, Synacthen®) to stimulate cortisol secretion [8]. It is recommended to use peak rather than the change in cortisol [11]. Screening can be conducted by determining a morning cortisol level; with a level <140 nmol/L combined with a 2-fold or greater raised ACTH level being suggestive of AI. The guidelines also recommend simultaneous measurement of plasma renin and aldosterone to determine the presence of mineralocorticoid deficiency [8].

The prevalence and characteristics of AI in bilateral adrenal metastases is not well-described. Thus, we have performed a systematic review and meta-analysis to collate all relevant articles in order to provide a summary on the topic.

## 2 Method

The systematic review was carried out as recommended by the PRISMA guidelines [12]. Systematic searches for eligible studies were conducted using PubMed/MEDLINE, ScienceDirect and Cochrane Reviews up to June 26, 2018, using the following strategy: "adrenal metastasis" AND ("adrenal failure" OR "adrenal insufficiency" OR "Addison disease" OR "Addison's disease" OR hypocortisolism) and "adrenal metastases" AND ("adrenal failure" OR "adrenal insufficiency" OR "Addison disease" OR "Addison's disease" OR hypocortisolism). In addition, the reference lists of the retrieved full-text studies were scanned to identify other potentially relevant studies. One further original research article known to us but not identified in the searches was added [13]. The relevance of the articles was first determined by reviewing the title, then the abstract, and if necessary, full-text retrieval. Two authors (PHT and HF) agreed on the included and excluded articles together with reference to the criteria described below. Articles were excluded if they were review articles or not in English.

Original research articles from the systematic review of the literature were used to collect cases of AI. To be included in the initial prevalence calculations, the cases had to meet the following criteria: bilateral adrenal disease confirmed by radiology or histology and having biochemical evidence of AI with an ACTH stimulation test. A second prevalence calculation was performed to extend the number of studies included which still required studies to demonstrate bilateral adrenal metastases but could use alternative means for the diagnosis of AI.

Individual cases of AI were excluded if they did not meet this criteria or if there was a nephrectomy, haemorrhage in an adrenal gland or treatment that contributed to the AI.

The individual cases of AI from original research articles were collated together with case reports to form a collection of cases of AI in Table 1. To be included, case reports needed to have documented bilateral disease and an ACTH stimulation test performed. If an ACTH test was not performed, then a baseline cortisol of <140 nmol/L was required to be consistent with diagnostic criteria from 2016 Guidelines [8]. Data was collected on age and sex of the patients, type of cancer, associated symptoms and signs of AI, biochemical features (including baseline and stimulated cortisol levels, and ACTH), size of metastases, treatment regimen and mortality. Mortality was recorded as time to death from the diagnosis of the AI.

**Table 1:** All published cases of bilateral adrenal metastasis and adrenal insufficiency.

First author	Age Sex	Cancer/Histology	Low BP	Post. low BP	Low Na	High K	Cortisol nmol/L (ug/dL)	ACTH pmol/L (pg/mL)	Peak cortisol after ACTH	Diagnosis	Largest size on CT (cm) left/right	Treatment and follow-up
Overt adrenal insufficiency												
Crisci *et al*[14]	69M	Colorectal	N	Y	-	-	220nmol (8ug/dL)	63 pmol/L (290 pg/mL)	-	ACTH stimulation test	-	TX with GC and MC. SX improved. Alive 12 months.
Cosentino *et al*[15],∗	43M	Lung ac	Y	Y	Y	Y	19 nmol/L (0.7ug/dL)	<2.2 pmol/L (<10 pg/mL)	-	Cortisol and ACTH	12L 12R	TX with GC and MC. Mortality - n/a
Imaoka *et al*[16]	82M	Colorectal	N	-	N	Y	215nmol (7.8ug/dL)	6.6 pmol/L (28 pg/mL)	499 nmol/L (18.1ug/dL)	ACTH stimulation test	2.5L 1.9R	TX with GC. Died 5 months later (unrelated to AI).
Mor *et al*[17]	43M	Colorectal	Y	-	Y	Y	408 nmol/L (14.8ug/dL)	114 pmol/L (520 pg/mL)	386 nmol/L (14.0ug/dL)	ACTH stimulation test	7L 9R	TX with GC. Died 8 months later.
Gul *et al*[36]	47F	Gastric ac	N	Y	Y	Y	275nmol (10ug/dL)	30.8 pmol/L (140 pg/dL)	-	ACTH-stimulation test	4.7L 3.8R	TX with GC. Mortality - n/a
Faulhaber *et al*[19]	69M	Lung signet ring cell ac	Y	-	Y	Y	28nmol (1.03ug/dL)	137.7 pmol/L (626 pg/mL)	-	Cortisol and ACTH	5L 5R	TX with GC and MC. SX resolved. Alive at 1 month
Noguchi *et al*[20]	78M	Small cell lung	Y	-	Y	Y	115 nmol/L (4.2ug/dL)	55.8 pmol/L (254 pg/mL)	77.2 nmol/L (2.8ug/dL)	ACTH-stimulation test	-	TX with GC. Died 4 months.
Bausewein *et al*[21]	75F	Breast	Y	-	Y	Y	148nmol (5.4ug/dL)	329 pmol/L (1499 pg/mL)	157.2 nmol/L (5.7ug/dL)	ACTH-stimulation test	-	TX with GC and MC. SX resolved. Mortality - n/a
Srinivasan *et al*[22]	71M	Melanoma	Y	-	Y	-	-	-	-	ACTH-stimulation test	9.6L 7.4R	TX with GC. SX resolved. Died 6 months later.
Goffman *et al*[23]	48M	Renal ca	Y	Y	Y	Y	504 nmol/L (18.3ug/dL)	-	675 nmol/L (24.5 ng/L)	ACTH-stimulation test	-	TX with GC. Died 3 days later.
Schnitzer *et al*[24]	74M	Lymphoma	-	Y	Y	-	22 nmol/L (0.8ug/dL)	-	-	ACTH-stimulation test	4L 4R	TX with GC. Died within 7 days.
Van den Heiligenberg *et al*[25]	78M	Non-hodgkin lymphoma	N	-	-	-	-	-	-	ACTH-stimulation test	5L 5R	TX with GC. SX improved. Died 3 weeks later.
Yeo *et al*[26]	78F	Breast	Y	-	-	-	265 nmol/L (9.6ug/dL)	-	235 nmol/L (8.5ug/dL)	ACTH-stimulation test	-	TX with GC. SX improved. Died 1 year later
Sheeler *et al*[27] Patient 1	52M	Lung ac	Y	Y	Y	Y	-	-	248 nmol/L (9ug/dL)	ACTH -stimulation test 40IU BD for 48 hrs	-	TX with GC. Died 6 months later.
Sheeler *et al*[27] Patient 2	75F	Breast	Y	N	Y	Y	182 nmol/L (6.6ug/dL)	80.3 pmol/L (365 pg/mL)	231 nmol/L (8.4ug/dL)	ACTH- stimulation test	-	TX with GC and MC. SX improved. Alive at 2 years.
Sheeler *et al*[27] Patient 3	65M	Small cell lung	Y	-	Y	Y	510 nmol/L (18.5ug/dL)	-	400 nmol/L (14.5ug/dL)	48hr ACTH infusion	8L 8R	TX with GC. Mortality - n/a
Sheeler et al [27] Patient 4	70M	Large cell lung	N	-	Y	Y	292 nmol/L (10.6ug/dL)	-	513 nmol/L (18.6ug/dL)	ACTH-stimulation test	3.5L 9R	TX with GC. SX improved. Mortality - n/a
Seidenwurm *et al*[29] Patient 1	51M	Lung	-	-	-	-	331 nmol/L (12ug/dL)	55 pmol/L (252 pg/mL)	358 nmol/L (13ug/dL)	ACTH-stimulation test	-	Responded to GC. Mortality - n/a
Seidenwurm *et al*[29] Patient 3	56M	Undifferentiated	-	-	-	-	259 nmol/L (9.4ug/dL)	-	303 nmol/L (11ug/dL)	ACTH-stimulation test	-	Responded to GC. Mortality - n/a
Seidenwurm *et al*[29] Patient 4	62M	Colon	-	-	-	-	-	-	-	ACTH-stimulation test	-	Responded to GC and MC. Mortality - n/a
Redman *et al*[30] Patient 1	-	-	-	-	-	-	303 nmol/L (11ug/dL)	-	331 nmol/L (12ug/dL)	ACTH-stimulation test	5L 5R	-
Redman *et al*[30] Patient 2	-	-	-	-	-	-	745 nmol/L (27ug/dL)	-	800 nmol/L (29ug/dL)	ACTH-stimulation test	2.5L 2.5R	-
Redman *et al*[30] Patient 3	-	-	-	-	-	-	469 nmol/L (17ug/dL)	-	524 nmol/L (19ug/dL)	ACTH-stimulation test	4L 3R	-
Redman *et al*[30] Patient 4	-	-	-	-	-	-	993 nmol/L (36ug/dL)	-	1020 nmol/L (37ug/dL)	ACTH-stimulation test	-	-
Redman *et al*[30] Patient 5	-	-	-	-	-	-	386 nmol/L (14ug/dL)	-	441 nmol/L (16ug/dL)	ACTH-stimulation test	3.5L 1.5R	-

Units Cortisol nmol/L (mg/dL); Units ACTH pmol/L (pg/mL); BP = blood pressure; Post. low BP = postural low blood pressure, i.e., orthostatic hypotensiont; Low Na = hyponatremia; High K = hyperkalemia; Y = yes; N = no; ' - ' = not available; GC = glucocorticoids; ac = adenocarcinoma; MC = mineralocorticoids; SX = symptoms; TX = treatment; n/a = not available.

∗ Cosentino *et al*. [15] - features of primary and secondary AI.

Articles arising from the literature search that were not included but contained data on adrenal function in malignancy or adrenal metastases without necessarily having AI were collected and summarised to provide some comparison of the collected data.

A quality assessment of the original research articles was incorporated with the strengths and limitations of each article noted in Table 2. Assessment using, e.g., the Newcastle-Ottowa Scale (www.ohri.ca/programs/clinical_epidemiology/oxford.asp), or any other standardized quality assessment instrument was considered inappropriate, as they were not developed to assess prevalence studies nor case reports/series.

**Table 2:** Quality assessment of prevalence studies included.

Author	Year	Type of Study	Strength	Limitations
Cedermark *et al.*[28]	1981	Retrospective observational study	• Histology proven adrenal metastases • Use of ACTH stimulation test for diagnosis with clear documentation of values. • Representative of patients with bilateral metastases	• Small sample size with 7 consecutive patients, 3 with bilateral adrenal metastases.
Seidenwurm *et al.*[29]	1984	Retrospective observational study	• Moderate sample size • Clear documentation of bilateral disease via CT or autopsy • Representative of patients with bilateral metastases	• Used symptoms, signs or biochemistry suggestive of AI, as well as response to glucocorticoids as part of diagnostic criteria rather than ACTH-stimulation testing (only 2 patients had documented inappropriate response to ACTH testing out of those included as AI) • May have included patients with nephrectomy/adrenalectomy
Redman *et al.*[30]	1987	Prospective observational study with intervention arm	• Excluded treatment induced AI • Used ACTH stimulation test for diagnosis with clear documentation of values • Clear documentation of bilateral disease • Representative of patients with bilateral metastases	• Small sample size
Lutz *et al.*[31]	2000	Cross sectional observation study.	• Clear documentation of bilateral adrenal disease • Exclude patients with prior treatment that could affect adrenal function. • Used ACTH stimulation testing • Representative of patients with bilateral disease	• Small sample size • Diagnostic criteria for subclinical AI not validated (use of ACTH:cortisol ratio)
Lam *et al.*[32]	2002	Retrospective observational study	• Large sample size • Clear documentation of bilateral disease via autopsy, adrenalectomy or FNA • Population representative of patients with bilateral metastases	• Inconsistent use of ACTH stimulation test for diagnosis • Authors did not disclose the values for diagnosis of AI • Diagnosed AI by using signs, symptoms and biochemical evidence with unclear documentation of what this entails.
Delivanis *et al.*[13]	2016	Retrospective observational study	• Moderate sample size with bilateral disease • Confirmed radiological evidence of bilateral disease • Representative of population of interest	• No ACTH stimulation testing, used low cortisol and high ACTH levels to diagnosis AI

AI = adrenal insufficiency, ACTH = adrenocorticotropin hormone, CT = computer tomography, FNA = fine needle aspiration.

All values for cortisol were documented in nmol/L (conversions made if articles reported values in ug/dL) and for ACTH levels, values were reported in pmol/L (conversions made for pg/mL).

Baseline serum/plasma cortisol at 8am was referred to as basal cortisol and a serum/plasma cortisol level after administration of ACTH (ACTH stimulation test) was referred to as stimulated cortisol.

Data from Table 3 of all collected cases of AI was used to assess certain outcomes. An outcome was reported as a percent of how many of the cases actually reported the outcome rather than total number of cases.

**Table 3:** Studies of prevalence of adrenal insufficiency in patients with bilateral adrenal metastases.

*Prevalence of AI as documented by ACTH stimulation test*			
Cedermark et al [28]	1981	0/4 (0%)	
Seidenwurm et al [29]	1984	2/57 (3.5%)	
Redman et al [30],∗	1987	5/15 (33.3%)	
Lutz et al [31],∗∗	2000	0/9 (0%)	
**Total prevalence of AI**			**7/85 (8.2%)**
**Total prevalence of AI excluding Redman et al**[30],*			**2/70 (2.9%)**
*Prevalence of AI as documented by either ACTH stimulation test or clinical criteria*			
Cedermark et al [28]	1981	0/4 (0%)	
Seidenwurm et al [29]	1984	5/57 (8.7%)	
Redman et al [30],∗	1987	5/15 (33.3%)	
Lutz et al [31],∗∗	2000	0/9 (0%)	
Lam et al [32]	2002	4/229 (1.74%)	
Delivanis et al [13]	2016	2/50 (4%)	
**Total prevalence of AI**			**16/264 (6.1%)**

∗ All cases had a basal cortisol level of >300 nmol/L but failed mount an appropriate response to ACTH stimulation.

∗∗ Lutz et al - study only contained subjects with subclinical AI rather than overt AI.

### 2.1 Statistical analysis

Results were calculated after pooling the results of all studies. Results are presented as the mean ± standard deviation (SD) or median and interquartile range (IQR) whichever was appropriate. Linear regression was used to evaluate the correlation between the size of adrenal metastases and cortisol and ACTH concentrations, respectively. Statistical significance was defined as P < 0.05. SigmaStat 3.0 for Windows (Systat Software Inc., San Jose, California) was used for all analysis.

## 3 Results

**Fig. 1:**
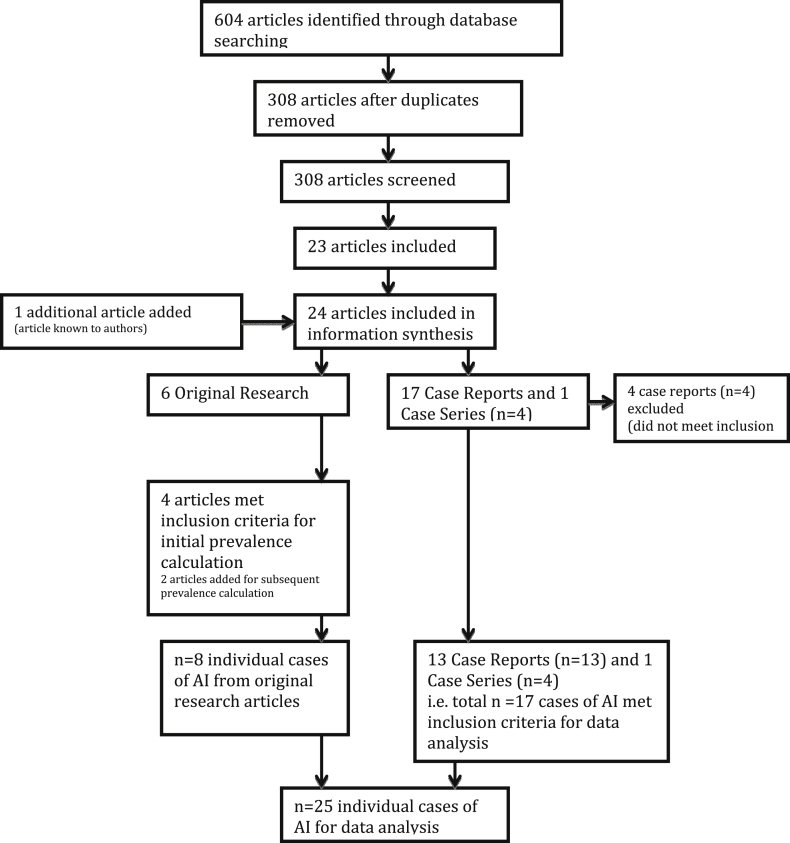
Flow diagram of process of systematic review.

The searches identified 604 articles of which 24 were considered eligible for inclusion in the systematic review (Fig. 1). Four case reports did not meet inclusion criteria leaving 13 cases reports [14, 15, 16, 17, 18, 19, 20, 21, 22, 23, 24, 25, 26], one case series of four patients [27] and six original research articles [13, 28, 29, 30, 31, 32]. Of these original research articles, four articles met criteria for the initial prevalence calculation requiring bilateral disease and ACTH stimulation test to diagnose AI. Six articles (an additional two [13, 32]) were used for the subsequent prevalence calculation which required less strict diagnosis of AI. All articles included in prevalence calculations are included in Table 3 and detailed comments on the quality of these studies are documented in Table 2. Two articles not meeting criteria had data included for comparison of cortisol between AI and non-AI groups as part of secondary outcomes [9, 33].

Of the 19 case reports detected by the search, 13 met inclusion criteria along with the case series, yielding a total of 17 individual cases of AI. Along with eight individual cases collected from the original research articles, there was a total of 25 cases of AI for data analysis which are tabulated in Table 1.

### 3.1 Primary outcome

#### 3.1.1 Prevalence of AI in bilateral adrenal metastases

A summary of the prevalence of AI in bilateral adrenal metastases is shown in Table 3. When using strict inclusion criteria of documented bilateral disease and an ACTH stimulation test for diagnosis, only four studies could be included [28, 29, 30, 31] which date from 1981 to 2000 giving a small sample size of 85. The prevalence of AI was 7/85 (8%) using this criteria. If excluding Redman et al. [30] since all 5 cases had a basal cortisol level of >300 nmol/L yet failed to mount an appropriate response to ACTH stimulation, the prevalence would be only 2/70 (3%). To expand the sample size, an additional two studies [13, 32] could be included yet they did not document an ACTH stimulation test as part of diagnostic criteria. This extended the date of included studies to 2016 and gave a sample size of 264. The prevalence of AI was then 16/264 (6.1%).

We have further described the studies below in chronological order of publication.

Cedermark et al [28] in 1981 documented seven patients with histology proven unilateral or bilateral adrenal metastases and evaluated their adrenal function. This was done with an ACTH stimulation test, of which the detailed criteria is not documented. Four patients had bulky bilateral adrenal metastases yet maintained normal adrenal function. When assessed histologically, the residual normal functioning adrenal tissue amounted to <20% of the total adrenal gland demonstrating the remarkable ability of the adrenal glands to maintain adrenal function with minimal functioning tissue.

Seidenwurm et al [29] in 1984 reported a case series of 6000 computed tomography (CT) scans, 949 autopsy reports (over period 1980–1981) and all endocrine referrals from 1964-1981. Patients had unilateral or bilateral adrenal metastases confirmed by biopsy or by CT. There were 21 CT scans, 35 autopsies and one endocrine referral with bilateral adrenal metastases (total n = 57). Criteria for diagnosis of AI was presence of two of the following: symptoms, signs or typical biochemistry suggestive of AI, low cortisol or poor response to ACTH test (defined as failure to increase cortisol above 551 nmol/L 1 hour after 250mcg of ACTH) or a therapeutic response to steroids. Eight patients met criteria for AI but three were excluded due to nephrectomy and only two included ACTH in their diagnosis of AI. Thus for our initial prevalence calculations of AI: 2/57 (4%) and our subsequent calculations 5/57 (9%).

Redman et al [30] in 1987 collected 15 consecutive patients with bilateral adrenal metastases confirmed on CT scan and performed baseline cortisol and ACTH stimulation tests on them. Of these 15 patients, 10 had lung cancer. AI was defined as failure to increase cortisol by 138 nmol/L above baseline or failure to have a stimulated cortisol >413 nmol/L. Five patients did not achieve this and were diagnosed with AI giving a prevalence of 5/15 (33%).

Lutz et al [31] performed a cross sectional study of 28 patients including controls and patients with either cancer, unilateral metastases or bilateral metastases. No patients had AI. It has been described in further detail below.

Two additional studies were added to expand the sample size [13, 32]. Lam et al [32] in 2002 performed an observational study of medical records over a 30 year period involving 229 patients (49% of their total number 464) with bilateral metastases confirmed on histology (via autopsy, fine needle aspiration (FNA) or adrenalectomy). Criteria for diagnosis of AI was signs, symptoms or biochemical evidence of AI, though whether an ACTH stimulation test was performed is not clear. Five patients met this criteria, however, one had pituitary involvement as well as adrenal involvement so was excluded. This left 4/229 patients with AI (2%).

Delivanis et al [13] in 2016 retrospectively analysed the medical records of 419 adrenal biopsies from 1994-2014 including clinical data, imaging and surgical management. Eighty-two patients had bilateral adrenal masses proven on imaging and of these, 50 were due to metastatic disease. Two of the 50 patients had primary adrenal insufficiency, though the diagnostic criteria for the AI is not clear. This gives a prevalence of 2/50 (4%).

### 3.2 Secondary outcomes

#### 3.2.1 Cortisol levels in patients with adrenal metastases with and without AI

The collected individual cases of AI with bilateral adrenal metastases from Table 1 were used to evaluate basal cortisol and stimulated cortisol. The systematic review also yielded five studies which commented on adrenal function in patients with and without AI (three studies already included in the review and two studies that did not meet inclusion criteria). These have been summarised in Table 4 and below.

**Table 4:** Summary of adrenal function in patients without AI in malignancy.

	Controls	Cancer, no metastases	U/L adrenal metastasis	B/L adrenal metastases but no AI	Disseminated malignancy	AI
*Baseline Cortisol*						
Lutz et al [31]	307.4 ± 33.2 nmol/L	477.5 ± 64.9 nmol/L	440.4 ± 53.5 nmol/L	637.6 ± 92.1 nmol/L	-	-
Redman et al [30]	-	-	-	582 nmol/L	-	656 nmol/L
Cedermark et al [9]	-	490 ± 24 nmol/L (well patients) 1044 ± 132 nmol/L	-	-	-	-
Cedermark et al [28]	-	-	-	862.5 nmol/L	-	-
Ross et al [33]	-	-	-	-	NSCLC 432 nmol/L SCLC 212 nmol/L	-
Individual cases of AI from Table 1	-	-	-	-	-	318 ± 237 nmol/L
*Stimulated cortisol or increase in cortisol*						
Lutz et al [31]	794.6 ± 41.2 nmol/L	939.7 ± 99.2 nmol/L	990.8 ± 92.9 nmol/L	1151.4 ± 155.5 nmol/L	-	-
Redman et al [30]	-	-	-	480 nmol/L (increase from baseline)	-	35 nmol/L (increase from baseline)
Cedermark et al [9]	-	930 ± 43 nmol/L (well patients) 1339 ± 117 nmol/L (unwell patients)	-	-	-	-
Cedermark et al [28]	-	-	-	1137.5 nmol/L (mean stimulated cortisol) 225 nmol/L (mean increase from baseline)	-	-
Ross et al [33]	-	-	-	-	828.5 (536–1675) nmol/L (median for both groups)	-
Individual cases of AI from Table 1	-	-	-	-	-	423 ± 238 nmol/L

U/L = unilateral; B/L = bilateral; NSCLC = non-small cell lung cancer; SCLC = small cell lung cancer.

**Fig. 2:**
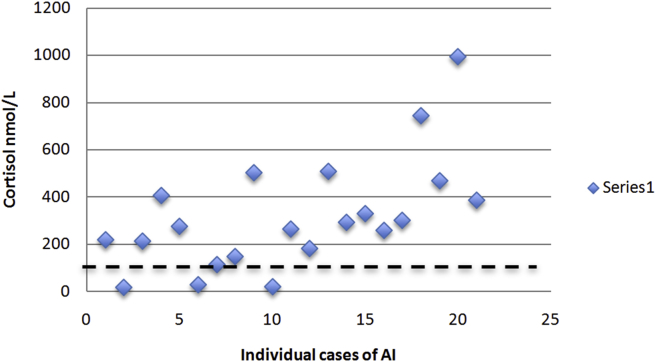
Basal cortisol concentrations (nmol/L) for individual cases of adrenal insufficiency due to bilateral adrenal metastases.

**Fig. 3:**
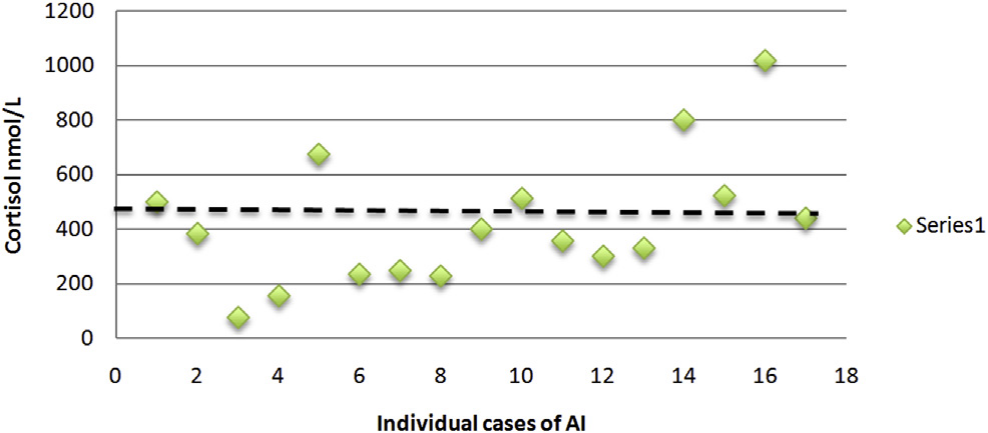
Stimulated cortisol concentrations (nmol/L) for individual cases of adrenal insufficiency due to bilateral adrenal metastases.

From our individual cases of AI (Table 1), the mean basal cortisol was 318 ± 237 nmol/L and the mean stimulated cortisol was 423 ± 238 nmol/L. The distribution of cortisol values have been plotted in Figs. 2 and 3 with a dashed line to demonstrated the number of cases with a basal cortisol over 140 nmol/L or a stimulated cortisol over 500 nmol/L.

When Redman et al [30] performed their study on 15 patients with bilateral metastases, we noted that the five patients with AI had baseline cortisol levels between 303 nmol/L and 717 nmol/L, higher than the criteria of <140 nmol/L. This study also compared the mean baseline cortisol for patients with and without AI and noted no significant difference (656 nmol/L versus 582 nmol/L, respectively). However, they noted a significant difference between the mean increase in cortisol with ACTH stimulation between AI and non-AI groups (increase by 35 nmol/L versus 480 nmol/L, respectively).

Lutz et al [31] similarly compared adrenal function amongst patients with malignancy where they performed a cross sectional study of 28 patients of whom none had AI on ACTH stimulation testing. There were seven normal controls, 11 patients with no metastases, eight with unilateral metastases and nine with bilateral metastases. Compared to controls (baseline cortisol 307.4 ± 33.2 nmol/L) the presence of cancer without metastases increased baseline cortisol to 477.5 ± 64.9 nmol/L, to 440.4 ± 53.5 nmol/L with unilateral metastases and to 637.6 ± 92.1 nmol/L for bilateral metastases. Similarly, for stimulated cortisol levels after ACTH administration there was a significant rise in levels with increasing degree of malignancy. Compared to controls (stimulated cortisol 794.6 ± 41.2 nmol/L) the presence of cancer without metastases increased levels to 939.7 ± 99.2 nmol/L; the presence of unilateral metastases increased levels to 990.8 ± 92.9 nmol/L; and bilateral metastases resulted in stimulated cortisol levels of 1151.4 ± 155.5 nmol/L.

Cedermark et al [28] recorded baseline and stimulated cortisol values for their four patients with bilateral metastases yet without AI. There was a mean baseline cortisol of 862.5 nmol/L and a mean stimulated cortisol 1137.5 nmol/L. The mean increase in cortisol from baseline was 225 nmol/L.

Additional studies that did not meet inclusion criteria for the systematic review but revealed additional data on adrenal function in malignancy were those of Cedermark et al [9] and Ross et al [33].

Cedermark et al [9] had a second study published in year 1981 that reviewed patients with cancer (non-lung cancer) that did not have adrenal metastases and evaluated their adrenal function via a cortisol and an ACTH stimulation test. They divided their patients into two groups - those that were in good physical condition (equivalent of Eastern Cooperation Oncology Group (ECOG) score 0–2) and those that were in poor physical condition (equivalent of ECOG 3 or 4). The first group (n = 24 patients), those in good physical condition, had a baseline cortisol of 490 ± 24 nmol/L and stimulated cortisol of 930 ± 43 nmol/L. Whereas, the second group (n = 15 patients), those in poor physical condition, had a much higher baseline cortisol of 1044 ± 132 nmol/L and stimulated cortisol of 1339 ± 117 nmol/L.

Ross et al [33] performed a prospective study on patients with lung cancer that was either non-small cell lung cancer (NSCLC) at stage 3 or 4 or small cell lung cancer (SCLC) at advanced stage. It is unclear if adrenal metastases were present or not but it was not part of their inclusion criteria. Adrenal function was assessed with baseline cortisol and an ACTH stimulation test which required a 30 minute cortisol to be > 550 nmol/L. In their two groups of NSCLC and SCLC, they noted median baseline cortisol was 432 nmol/L for NSCLC and 212 nmol/L for SCLC. The median peak cortisol at 30 minutes for both groups combined was 828.5 nmol/L. Interestingly, they noted two patients with a stimulated cortisol less than their 550 nmol/L criteria (536 nmol/L and 545 nmol/L, respectively) which was thought to be consistent with AI. However, because no documentation of definite adrenal involvement could be gleaned from the study results, these could not be included in our calculations.

#### 3.2.2 Other secondary outcomes

Basal ACTH levels in the collected cases of AI revealed median ACTH concentration of 59 pmol/L (IQR 31–114). In terms of clinical features, 11/16 (68.8%) were hypotensive, 13/14 (9.3%) were hyponatremic and 12/12 (100%) were hyperkalemic. Lung cancer was the most common malignancy reported to cause AI with 35% (7/20) of cases due to lung cancer. This was followed by colorectal 20% (4/20), breast cancer 15% (3/20) and lymphoma 10% (2/20).

The size of the adrenal metastases was 5.5 ± 2.8 cm (left) and 5.5 ± 3.1 cm (right), respectively. The total size of the largest diameter of both the left and right adrenal metastases was 11.0 ± 5.6 cm. There was no correlation between basal or stimulated cortisol concentration and the size of adrenal metastases (r = -0.298, P = 0.323 and r = -0.465, P = 0.207, respectively) nor ACTH concentrations and the size of adrenal metastases (r = 0.0622, P = 0.921).

Of those with documented treatment (80% of cases), 70% (14/20) used glucocorticoids alone and 30% (6/20) used a combination of glucocorticoids and mineralocorticoids. In those cases where mortality had been noted, the median time to death was 5.0 months (IQR 0.6–6.5). However, there were two cases being alive 12 months after diagnosis.

## 4 Discussion

This is the first systematic review summarising data on AI in patients with bilateral adrenal metastases. We provide data on prevalence, etiology, clinical features, diagnosis and mortality.

The prevalence of primary AI in bilateral adrenal metastases (ideally diagnosed with ACTH stimulation testing) was 3–8% or 6.1% if allowing for more lax diagnostic criteria (as per secondary calculations). This systematic review demonstrated the paucity of good quality studies on the topic and a need for further research with consistent diagnostic criteria of AI in the setting of bilateral adrenal metastases. This is important when reflecting on our prevalence calculations.

Despite the limited number of studies included, there is valuable information to be gleaned regarding adrenal function in malignancy. First of all, basal cortisol levels in cases of AI demonstrate that majority of cases are much higher than 140 nmol/L and supports its unreliability as a screening tool for patients with malignancy. This is likely due to the "stressed" adrenal response that results from advanced malignancy. As consistent with recommended diagnostic criteria, the stimulated cortisol level is more helpful in distinguishing AI from non-AI patients as seen in Fig. 3 and the study by Redman et al [30].

This review yielded data supporting the ability of the adrenal gland to maintain function until most of the gland is destroyed (>90%) [28], an important concept to remember when faced with large bilateral adrenal metastases on radiology. We also had data on the adrenal gland function as malignancy progressed from focal disease to metastatic to involving one or both adrenal glands. There was a consistent rise in basal and stimulated cortisol levels for each step in the progression of malignancy, which may or may not be relevant when it comes to performing the ACTH stimulation test in each patient group. Even the functional performance of a patient (i.e. ECOG status) reflects the underlying "stress" on the adrenal system [9]. On the other hand, severe weight loss can reduce plasma corticosteroid binding globulin, which if not met by an increase in cortisol secretion may lower measured total plasma cortisol [34]. How the ACTH stimulation test performs as a screening and diagnostic tool in patients with AI due to adrenal metastases compared to AI due to other causes is unclear but should be investigated in future studies.

Screening all patients with bilateral adrenal metastases for AI with ACTH stimulation testing is possible, although the cost-benefit of this approach has not been evaluated, especially as the presence of AI can be excluded clinically in some patients, e.g., in those with hypertension, and the detection of mild AI may not influence prognosis. However, symptoms attributed to advanced malignancy or cancer therapy may be the unrecognised presentation of AI and a clinical assessment in patients with bilateral metastases with selected ACTH stimulation testing seems reasonable.

Minimal focus has been played on use of ACTH in assisting diagnosis of AI in the setting of malignancy. However, as Cedermark et al [9] pointed out that lung malignancies may release an ACTH-like substance and the fact that many of the cases of AI in this review were due to lung cancer, ACTH may not be optimal as a screening tool for AI.

This systematic review and meta-analysis provides information on clinical features of AI in the cohort of patients with bilateral adrenal metastases. It was common to see the classic clinical and biochemical features of hypotension, hyponatremia and hyperkalemia, however, majority of these were from case reports where patients presented with overt adrenal failure. A number of studies [9, 29, 30, 31, 33] commented that patients not meeting criteria for AI had symptoms and signs of AI suggesting its lack of utility and significance in metastatic malignancy.

In regards to size of adrenal metastases on radiology, there was large variation and no meaningful information can be gleaned from the small number of cases, and there were no significant correlations between cortisol nor ACTH concentrations and the total size of the adrenal metastases. Further studies with comparison of size between AI and non-AI patients with bilateral metastases is required.

There has been a paucity of detailed data on treatment and survival for patients with AI in malignancy. Along with overlap of clinical signs, there is treatment overlap between the two conditions. Many patients with malignancies are treated with glucocorticoids to alleviate nausea from chemotherapy, reduced cerebral oedema and for its lymphocytolytic effects [35]. Thus, a clinical response to treatment with glucocorticoids is not specific to AI. However, in those cases included with documented follow-up the prognosis was very poor.

This systematic review and meta-analysis had limitations, the largest being the small number of studies meeting inclusion criteria for prevalence calculations as well as the small number of individual cases. Majority of the original research articles were published over 20 years ago. Even though an ACTH stimulation test was required for inclusion, the cortisol levels for diagnosis varied between studies. Moreover, majority of studies were retrospective observational studies and the inherent limitations of all retrospective studies, in particular that of ascertainment bias, were also present in this systematic review. Selection bias may have driven those tested for cortisol levels, especially when performed on unstimulated samples, leading to overestimation of AI prevalence.

Most case reports were in overt adrenal failure with obvious clinical signs and biochemical features (most had the classic hyponatremia and hyperkalemia with hypotension), which may reduce the generalization to a broader population of patients with bilateral adrenal metastases. A lack of reporting of detailed clinical features, in particular survival duration, of the identified cases limited clinical analyses.

A comprehensive study of this area would involve detection of adrenal metastases on routine abdominal imaging, followed by prospective assessment for symptoms of AI, an ACTH-stimulation test documenting values for those with AI and without AI, baseline ACTH, measures of aldosterone and renin, followed by an assessment of response to treatment after two weeks of hormone replacement and longer term follow up of survival between the two groups.

## 5 Conclusion

Adrenal insufficiency, variously defined by different studies, occurs in approximately 3–8% of individuals with bilateral adrenal metastases. AI was most commonly seen in bilateral adrenal metastases from lung cancers. The effects of malignancy on cortisol levels was notable for increasing stages of metastasis and its effect on interpretation and utility of the ACTH stimulation test is unknown. Of note, baseline cortisol, ACTH, clinical signs or symptoms of AI or response to steroids play limited role in diagnosis or screening for AI in patients with bilateral adrenal metastases. Physicians should have an increased awareness of the possibility of AI in malignancy and rely on ACTH stimulation testing for diagnosis. Further studies of patients with bilateral adrenal metastases with and without AI are required.

## Declarations

### Author contribution statement

Philippa H Tallis: Conceived and designed the experiments; Performed the experiments; Analyzed and interpreted the data; Contributed reagents, materials, analysis tools or data; Wrote the paper.

R. Louise Rushworth, David J Torpy: Contributed reagents, materials, analysis tools or data; Wrote the paper.

Henrik Falhammar: Conceived and designed the experiments; Analyzed and interpreted the data; Contributed reagents, materials, analysis tools or data; Wrote the paper.

### Funding statement

This work was supported by Magnus Bergvall Foundation, Sweden, Grant nr 2018-02566.

### Competing interest statement

The authors declare no conflict of interest.

### Additional information

No additional information is available for this paper.
